# Comparative study of serum zinc concentrations in benign and malignant prostate disease: A Systematic Review and Meta-Analysis

**DOI:** 10.1038/srep25778

**Published:** 2016-05-12

**Authors:** Jiang Zhao, Qingjiang Wu, Xiaoyan Hu, Xingyou Dong, Liang Wang, Qian Liu, Zhou Long, Longkun Li

**Affiliations:** 1 Department of Urology, Second Affiliated Hospital, Third Military Medical University, Chongqing, 400037, China

## Abstract

Many studies have investigated the relationship between serum zinc concentration and prostatic disease, but have shown inconsistent results. Hence, we performed a systematic literature review and meta-analysis to assess the correlation between serum zinc concentration and prostate disease. Systematic literature searches were conducted with PubMed, EMBASE, Science Direct/Elsevier, MEDLINE, CNKI and the Cochrane Library up to June 2015 for studies that involved the relationship between serum zinc concentration and prostate disease. Fourteen studies were identified from the databases. Our results illustrated that the serum zinc concentrations in prostate cancer patients were significantly lower than those in Benign prostatic hyperplasia (BPH) patients and normal controls (SMD (95% CI), −0.94 [−1.57, −0.32]; −1.18 [−1.90, −0.45]). However, the serum zinc concentrations in BPH patients were significantly higher than those in normal controls (SMD (95% CI) 1.77 [0.15, 3.39]). The present study showed that different levels of serum zinc concentrations are correlated with different prostatic disease. Serum zinc concentration may be used as a tool for the diagnosis and screening of prostate disease. But, further studies with well-designed larger sample studies are needed in this field to further clarify the correlation between serum zinc concentration and prostate disease.

Zinc is an important trace element that is relatively nontoxic and plays a very important role in human metabolism. Zinc has been found to be most highly concentrated in the liver, kidney, retina, prostate, and muscle^1^,^2^. Prostatic androgen metabolism is modified by the intracellular concentration of Zinc. At high tissue concentrations, this trace element inhibits the transformation of testosterone to dihydrotestosterone and plays an important role in maintaining the physiological function and normal tissue structure of the prostate^1^,^2^,^3^.

Zinc content varies in different organs and is highest in the prostate. Zinc mainly accumulates in the area surrounding prostate epithelial cells. Its content in the prostate is 100 times greater than that in plasma. The high concentration of zinc in the prostate strongly suggests that zinc plays an important role in prostate health. Some studies have reported that the Zn concentration in prostatic tissue is markedly decreased in prostate carcinoma patients and is increased in BPH patients^3^,^4^,^5^,^6^,^7^.

Serum zinc concentration is an appropriate biomarker of zinc status, as it has been confirmed to respond to zinc intake and to correctly predict functional responses to zinc interventions^8^. Zinc concentration is much easier to measure in serum than in the prostate due to the ease of access to serum and can be obtained without causing prostatic damage. Information regarding serum zinc concentration is of obvious interest to improve our understanding of the etiology and pathogenesis of prostatic diseases and also to aid in their diagnosis. However, information about serum zinc concentration in prostatic diseases is lacking and research findings have been highly contradictory.

Studies have investigated the correlation between serum zinc concentration and prostate disease, but their results have been contradictory, with some studies showing a statistically significantly lower serum zinc concentration in prostate cancer patients than in normal controls and others not observing this phenomenon. Several studies have shown that serum zinc concentration in BPH patients is statistically significant higher than that in normal controls, but others did not observe this phenomenon^9^,^10^,^11^,^12^,^13^,^14^,^15^,^16^,^17^,^18^,^19^,^20^,^21^,^22^. Therefore, we systematically reviewed the available literature and performed a meta-analysis to evaluate the correlation between serum zinc concentration and prostate disease to obtain valuable insight into the diagnosis and treatment of prostatic disease.

## Results

### Characteristics of the included studies

**Figure 1:**
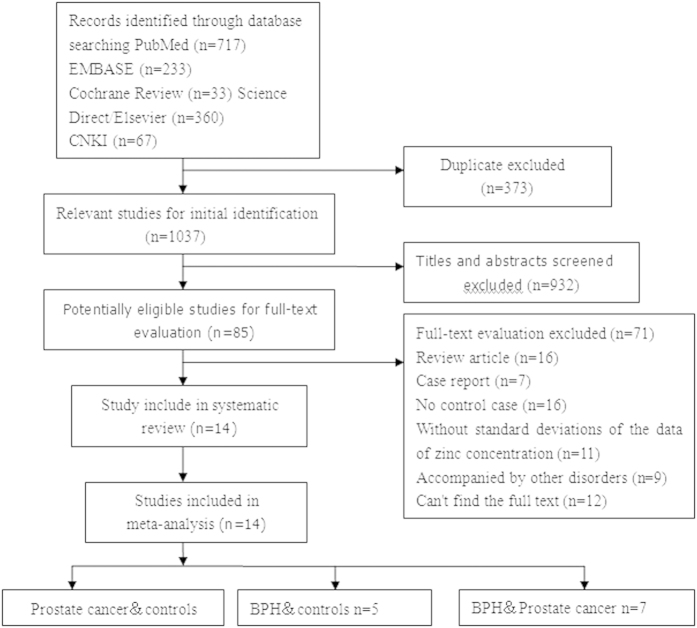
Flow diagram of selection of eligible studies.

Figure 1 shows the review process in detail. A total of 1037 unduplicated studies were identified, fourteen studies were ultimately selected according to the eligibility criteria. Ten studies investigated the correlation between serum zinc concentration and prostate cancer. Five studies investigated the correlation between serum zinc concentration and BPH, and seven studies investigated the correlation of serum zinc concentration between prostate cancer and BPH. After group discussion, all reviewers were in agreement to include all twelve papers.

Table 1 summarizes the general data from the fourteen studies. The retrieved studies included a total of 1318 patients with prostate disease and 1413 normal controls. The mean ages of the prostate cancer, BPH and control groups were in the ranges of 59.3–73.6 years, 63.4–76.8 years and 65.9–74.2 years, respectively. The mean ages of the patients and control groups were unavailable for three studies^13^,^16^,^18^. All studies reported exclusion/inclusion criteria^9^,^10^,^11^,^12^,^13^,^14^,^15^,^16^,^17^,^18^,^19^,^20^,^21^,^22^. For the ten studies^9^,^10^,^11^,^12^,^13^,^14^,^15^,^16^,^21^,^22^ that investigated the correlation between serum zinc concentration and prostate cancer, a total of 794 prostate cancer cases and 1359 normal controls were included. For the five studies^11^,^12^,^13^,^16^,^17^ that investigated the correlation between serum zinc concentration and BPH, a total of 194 cases and 333 normal controls were included. For the seven studies^11^,^12^,^13^,^16^,^18^,^19^,^20^ investigated the difference in serum zinc concentration between prostate cancer and BPH patients, a total of 336 prostate cancer patients and 337 BPH patients were included.

### Meta-analysis

**Figure 2:**
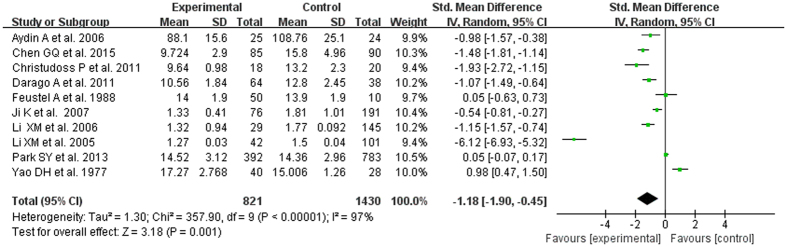
Forest plot showing the meta-analysis outcomes of the correlation between serum zinc concentration and prostate cancer.

**Figure 3:**
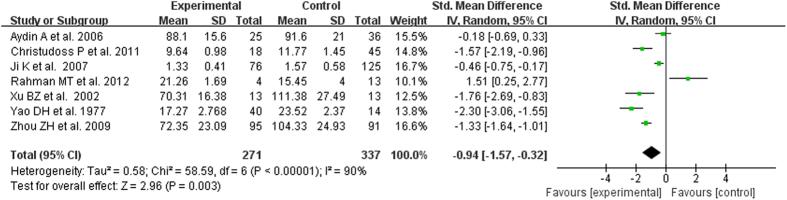
Forest plot showing the meta-analysis outcomes of the difference in serum zinc concentration between prostate cancer and BPH patients.

**Figure 4:**
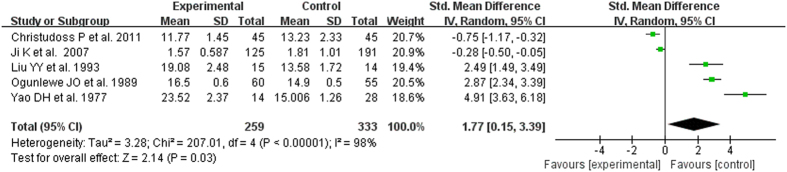
Forest plot showing the meta-analysis outcomes of the correlation between serum zinc concentration and BPH.

**Figure 5:**
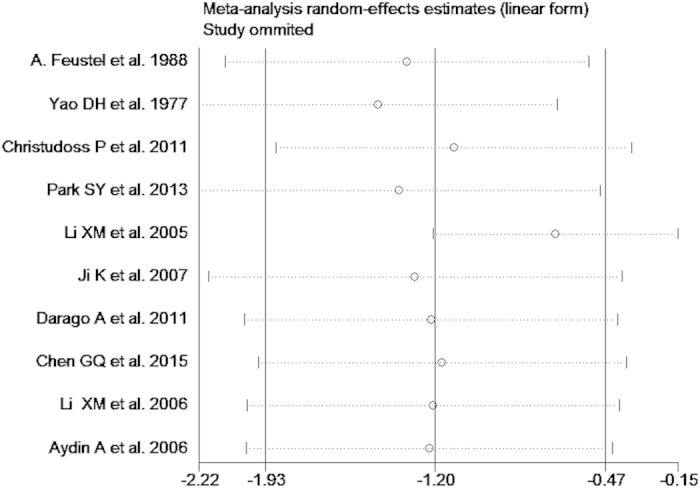
Sensitivity analysis plot of serum zinc concentration and prostate cancer.

**Figure 6:**
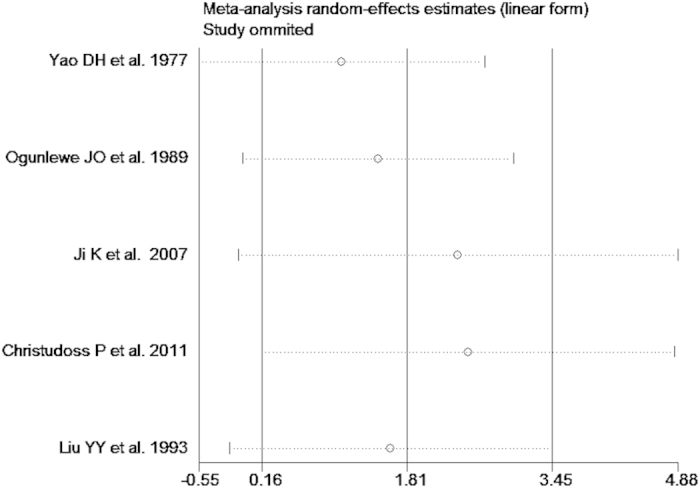
Sensitivity analysis plot of serum zinc concentration and BPH.

**Figure 7:**
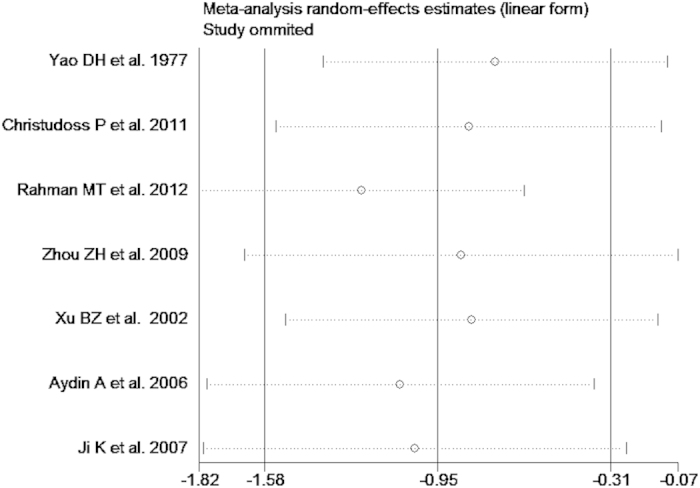
Sensitivity analysis plot of serum zinc concentration in prostate cancer and BPH.

The test of heterogeneity suggested a random effects model, and the meta-analysis revealed that the serum zinc concentration of prostate cancer patients was significantly lower than that of normal controls and BPH patients (SMD (95% CI), −0.94 [−1.57, −0.32]; −1.18 [−1.90, −0.45]) (Figs 2 and 3). However, the serum zinc concentration of BPH patients was significantly higher than that of normal controls (SMD (95% CI) 1.77 [0.15, 3.39]) (Fig. 4). Egger’s regression test indicated little evidence of publication bias (Prostate cancer & controls: t = −2.26 and p = 0.06 > 0.05; BPH & controls; t = 2.07 and p = 0.13 > 0.05; BPH & Prostate cancer: t = −0.21 and p = 0.84 > 0.05) (Table 2). We also conducted a sensitivity analysis of the meta-analysis. We omitted each study sequentially, and the calculated combined SMDs for the remaining studies yielded consistent results. No single study significantly altered the combined results of the overall meta-analysis, which indicated that the results were statistically stable and reliable (Figs 5,6 and 7).

## Discussion

In our study, fourteen literatures studied the correlation between serum zinc concentration and prostate disease^9^,^10^,^11^,^12^,^13^,^14^,^15^,^16^,^17^,^18^,^19^,^20^,^21^,^22^. Ten of the fourteen literatures^9^,^10^,^11^,^12^,^13^,^14^,^15^,^16^,^21^,^22^ studied the relationship between the serum zinc concentration and prostate cancer. Seven studies^11^,^12^,^13^,^14^,^15^,^21^,^22^ reported that the zinc concentration of prostate cancer patients was significantly lower than that of normal controls, while two studies showed a non-statistically significant difference^9^,^10^. One study^16^ reported that the zinc concentration of prostate cancer patients was significantly higher than that of normal controls. In this meta-analysis, the zinc concentration of prostate cancer patients was significant lower than that of normal controls (SMD (95% CI), −0.94 [−1.57, −0.32]). Five literatures^11^,^12^,^13^,^16^,^17^ studied serum zinc concentration in BPH patients; three of those studies^16^,^17^,^23^ reported that the zinc concentration of BPH patients was significant higher than that of normal controls, while two studies^12^,^13^ reported that the zinc concentration of BPH patients was significantly lower than that of normal controls. In our study, the zinc concentration of BPH patients was significantly higher than that of normal controls (SMD (95% CI), −1.18 [−1.90, −0.45]). Seven literatures^11^,^12^,^13^,^16^,^18^,^19^,^20^ studied the relationship of serum zinc concentration between prostate cancer and BPH patients. Five studies^12^,^13^,^16^,^19^,^20^ reported that the serum zinc concentration in prostate cancer patients was significantly lower than that in BPH patients, and one study^18^ reported a contrasting result. In this meta-analysis, the serum zinc concentration in prostate cancer patients was significant lower than that in BPH patients (SMD (95% CI), 1.77 [0.15, 3.39]).

Zinc has an important role in human prostatic physiological metabolism. Currently, the understanding of the role of zinc in the prostate are as follows: a. maintenance of physiological function and normal tissue structure (this is especially important for the maintenance of the integrity and stability of the acinar epithelium and ductal epithelium); b. affect the activity of enzymes by acting as a coenzyme, with changes in its concentration greatly influencing the activities of many enzymes^1^,^24^,^25^; c. maintenance of the stability of sperm chromatin in seminal plasma, as indicated by the fact that healthy persons with high zinc content in the seminal plasma had a high percentage of sperm with stable chromatin, while infertile patients had lower zinc content in the seminal plasma and a lower percentage of sperm with stable chromatin^26^,^27^; d. killing of common bacteria that cause urogenital infection; e. involvement in the regulation of the growth and apoptosis of prostate epithelial cells^28^,^29^. Studies have reported that zinc may be protective against the development and progression of prostate cancer and may be benefit in the patients of with chronic prostatitis^30^,^31^,^32^,^33^,^34^,^35^,^36^,^37^,^38^,^39^. In prostate cancer, Zinc influences the development of prostate carcinoma. Currently, studies have shown that zinc inhibits prostate carcinoma cell growth, possibly due to the induction of cell-cycle arrest and apoptosis^25^,^40^,^41^. In general, the healthy human prostate accumulates the highest level of zinc in prostate epithelial cells, but this property is lost in prostatic malignancy^42^,^43^,^44^. Indeed, the highest level of zinc in prostate cells diminish early in the course of prostate carcinogenesis, preceding histopathological changes, and continue to decline during progression toward castration-resistant disease^25^,^40^,^41^,^42^,^43^,^44^. In this study, serum zinc concentrations in prostate cancer patients were significantly lower than those in Benign prostatic hyperplasia (BPH) patients and normal controls. This results suggest that serum zinc concentrations diminish may be attenuated prostate accumulates the highest level of zinc and promote tumor development in prostate cancer patients. Oppositely, Some studies have reported that higher concentration of zinc may be the adverse effect of BPH^43^,^45^,^46^,^47^,^48^. The serum zinc concentration of BPH patients was significantly higher than that of prostate carcinoma patients and normal controls. This finding contrasts with that in prostate carcinoma, and the mechanism behind the effects of zinc in BPH are not clear. Potentially, Zinc induces a bell-shaped proliferative of smooth muscle cells from benign prostatic hyperplasia^48^.

The potential mechanisms which zinc concentrations increased in BPH but decreased in prostate cancer is unclear. Serum zinc level variety is a consequence of nutritional status, dietary, tumor, chronic stress, chronic diseases, aging or of a combination of all these effects^30^,^44^,^49^. Chronic stress and inflammation is a common hallmark of cancer could lead to redistribution of zinc between body compartments which zinc importer (ZIP), zinc exporter (ZnT), and metallothionein may be involved and thus may be important mechanism of serum zinc level decrease^30^,^41^,^42^,^44^,^49^. Previous studies have shown that nutritional status, diet, race and clinical conditions may also influence the serum zinc concentration^1^,^49^,^50^,^51^,^52^. In our study, clinical conditions, nutritional status, diet and race of patients may be a crucial factor in serum level of zinc. In this study, the serum zinc concentration of patients with both prostatic carcinoma and metastases was decreased in comparison to control and BPH^9^,^10^,^12^,^13^,^14^,^15^,^16^,^23^. But, in this meta analysis, due to the reasons for the research design form the published literature, study on correlation between serum zinc concentration with tumor grade, stage and histological type is unable to assess. In addition, in this meta analysis, we included different countries and races of patients^9^,^10^,^11^,^12^,^13^,^14^,^15^,^16^,^21^,^22^. This will lead to different diet habits, nutritional status and lifestyle. These factors may be affect the serum zinc concentration of the included patients. But, in this meta analysis, because the research design and small sample size form the published literature, study on correlation between serum zinc concentration with nutritional status, diet and race is still unable to assess. Therefore, further studies with a larger sample of well-designed studies are needed to illuminate the relationship between serum zinc concentration and nutritional status, diet, race and clinical conditions.

There are some limitations that need to be taken into consideration when interpreting the results of this meta-analysis. First, the sample sizes of all included studies were relatively small, with a total of 1318 disease patients and 1413 normal controls in all twelve studies. Secondly, several studies related to the subject of interest were excluded due to a lack of control data (means and standard deviations) or unavailability of the full text. Thirdly, although this meta-analysis showed that serum zinc concentration was decreased in prostate cancer patients and increased in BPH patients, it was not clear whether prostatic disease caused the change in serum zinc concentration or the change in the serum zinc concentration led to prostatic disease. The result are not sufficient to evaluate causality. Fourth, serum zinc concentration has a limited predictive value because it is a specific intracellular ion that fluctuates with the circadian rhythm^53^. Therefore, it is necessary that through averaging the testing values of repeated measurements and precise sampling time at 8.00 which may be the best time. Therefore, this meta-analysis just access the real results of the zinc and prostate disease, and this phenomenon may benefit for the diagnostic of prostate disease, but further studies is warranted to confirm these findings.

In summary, the present study showed that serum zinc concentration was significantly lower in prostate cancer patients than in normal controls. Additionally, serum zinc concentration was significantly higher in BPH patients than in prostate cancer patients and normal controls. Serum zinc concentration may be used as a tool for the diagnosis and screening of prostate disease. But, further studies with well-designed larger sample studies are needed in this field to further clarify the correlation between serum zinc concentration and prostate disease.

## Methods

### Literature search

This meta-analysis was restricted to published studies that investigated the difference in serum zinc concentration between prostatic disease patients and normal controls. Two independent reviewers searched PubMed, EMBASE, Science Direct/Elsevier, Medline CNKI and the Cochrane Library from inception to June 2015, without language or study type restriction. The search terms combined text words and MeSH terms. For example, the search terms for serum zinc concentration were ‘serum zinc concentration’, ‘serum zinc content’, ‘zinc concentration’, ‘zinc content’, ‘serum zinc level’, ‘zinc level’, ‘zinc status’ and ‘trace element’ those for prostatic cancer and BPH were ‘prostatic cancer’, ‘prostatic tumour’, ‘prostate malignancies’, ‘prostate carcinoma’, ‘Prostate malignant diseases’, ‘Benign Prostatic Hyperplasia’, ‘BPH’, ‘prostatic benign diseases’, and ‘prostatic hyperplasia’. All related articles and abstracts were retrieved. In addition, references cited within the relevant articles and abstracts were retrieved manually, but only references that were full articles were reviewed for eligibility.

### Eligibility criteria

#### Inclusion criteria

Studies were included their study population included patients diagnosed with prostatic carcinoma and benign prostatic hyperplasia. The controls were healthy human males with no history or evidence of andrologic or urologic disease. Zinc was detected in the serum by the atomic absorption spectrophotometry (AAS) test. Available data were extracted from the eligible articles, including means and standard deviations of serum zinc concentrations in all case-control groups.

#### Exclusion criteria

Studies were excluded if they were case reports or review articles. Studies involving patients with prostatic disease accompanied by other disorders of the urogenital system, prostate metastatic carcinoma patients, and patients who were ongoing zinc supplementation therapy were also excluded.

### Study selection and validity assessment

Two independent reviewers screened the titles and abstracts of all citations from the literature search. All relevant studies that appeared to meet the eligibility criteria were retrieved. If it was unclear whether the study was eligible for inclusion based on the review of the title and abstract, the full text was examined. The full texts of all potentially eligible studies were reviewed to confirm their eligibility. Disagreements were resolved by consensus or by a third reviewer. Two reviewers completed quality assessments of the included studies according to the primary criteria of the Newcastle-Ottawa Quality Assessment scale (NOS) for assessing the quality of nonrandomized and observational studies in meta-analyses.

### Data extraction and statistical analysis

Data, including demographic data (authors, year of publication, country, number and mean age of participants, and assay method) and the outcome data of serum zinc concentration in all case-control studies were extracted by three reviewers. Disagreements were resolved by consensus. A quantitative meta-analysis was performed by two reviewers using Review Manager (RevMan) software (version 5.2, The Nordic Cochrane Centre, The Cochrane Collaboration, 2012, Copenhagen) and Stata software (version 12.0, College Station, Texas, USA). Available data were analyzed in the meta-analysis.

We pooled the standard mean differences (SMD) of the serum zinc concentrations of the case-control groups, which were identified with 95% confidence intervals (95% CI). Heterogeneity was assessed by the P-value and the I-square statistic (I^2^) in the pooled analyses, which represent the percentage of total variation across studies. If the P-value was less than 0.1 or the I^2^-value was greater than 50%, the summary estimate was analyzed by a random-effects model. Otherwise, a fixed-effects model was applied. To estimate the stability of the Meta-analysis, we conducted a sensitivity analysis. In addition, publication bias was detected by the Egger’s test, and a P-value of less than 0.05 indicated the presence of publication bias.

## Additional Information

**How to cite this article**: Zhao, J. *et al.* Comparative study of serum zinc concentrations in benign and malignant prostate disease: A Systematic Review and Meta-Analysis. *Sci. Rep.* **6**, 25778; doi: 10.1038/srep25778 (2016).

## Figures and Tables

**Table 1:** Characteristics of include studies investigating the serum zinc and prostatic disease.

Study	Country	Mean age (Cancer/BPH/control)	Control (n)	Cancer	BPH	Assay
Feustel A 1989	German	68.6/65.9/NI	10	50		AAS
Park SY 2013	America	68.9 ± 7.2/69.1 ± 7.1/NI	783	392		AAS
Aydin A 2006	Turkey	65.0 ± 6.0/64.3 ± 7.9/67.5 ± 8.8	24	25	36	AAS
Ji K 2007	China	70.91 ± 7.99/68.63 ± 7.53/69.11 ± 7.87	191	76	125	AAS
Christudoss P 2011	India	NI/NI/NI	20	18	45	AAS
Li XM 2006	China	72 ± 1.62/71.5 ± 0.6/NI	145	29		AAS
Daragó A 2011	Poland	59.30 ± 5.08/70.18 ± 6.30/74.25 ± 5.40	20	10		AAS
Yao DH 1977	China	NI/NI/NI	28	40	14	AAS
Liu YY 1993	China	64/65/28	14		15	AAS
Rahman MT 2012	Bangladesh	NI/NI/NI		5	13	AAS
Xu BZ 2002	China	71.9/72.2/NI		13	13	AAS
Zhou ZH 2009	China	73.6/71.5/NI		95	91	AAS
Li XM 2005	China	71.9/72.2/NI	38	64		AAS
Chen J.Q 2015	China	65.9 ± 8.4/76.8 ± 12.5/NI	85	90		AAS
Ogunlewe J.O 1989	Nigeria	61.2 ± 6.9/67.3 ± 8.0/65.8 ± 8.1	55		60	AAS

Abbreviations: NI not indicated in studies. AAS, atomic absorption spectrophotometry.

**Table 2:** The Egger’s test of Publication bias.

	Coef.	Bias Std. Err.	t	P > |t|	[95% Conf.Interval]	
Prostate cancer & controls	−6.35	2.80	−2.26	0.06	−12.82	0.13
BPH & controls	9.50	4.60	2.07	0.13	−5.12	24.12
BPH & Prostate cancer	0.61	2.97	0.21	0.84	8.23	6.99
