# Erratum: Comparative study of serum zinc concentrations in benign and malignant prostate disease: A Systematic Review and Meta-Analysis

**DOI:** 10.1038/srep28606

**Published:** 2016-07-20

**Authors:** Jiang Zhao, Qingjian Wu, Xiaoyan Hu, Xingyou Dong, Liang Wang, Qian Liu, Zhou Long, Longkun Li

Scientific Reports 6: Article number: 2577810.1038/srep25778; published online: 05122016; updated: 07202016

The original version of this Article contained a typographical error in the spelling of the author Qingjian Wu, which was incorrectly given as Qingjiang Wu. This has now been corrected in the PDF and HTML versions of the Article.

